# Is there a transcatheter solution for a sick neonate with hypoplastic right heart syndrome?: Pulmonary valve perforation in a neonate with hypoplastic right ventricle with pulmonary atresia, restrictive VSD—a case report

**DOI:** 10.1186/s43044-020-00097-7

**Published:** 2020-09-29

**Authors:** Parag Barwad, Krishna Prasad, Jyothi Vijay, Sanjeev Naganur

**Affiliations:** Department of Cardiology, Advanced Cardiac Centre, Post Graduate Institute of Medical Education & Research, Sector 12, Chandigarh, 160 012 India

**Keywords:** Hypoplastic Right heart syndrome, Pulmonary atresia, Pulmonary valve perforation, Case report, Cyanotic heart disease, Duct dependent

## Abstract

**Background:**

Hypoplastic right heart syndrome with pulmonary atresia is a rare cyanotic heart disease with poor prognosis requiring urgent intervention to establish the pulmonary blood flow. Pulmonary blood flow is achieved by BT shunt or percutaneous techniques like PDA stenting or pulmonary valve perforation. Various series have shown that early surgical intervention causes high mortality in these patients. Pulmonary valve perforation is a suitable, physiological alternative to surgical techniques in selected patients.

**Case presentation:**

We report a case of hypoplastic right heart syndrome with pulmonary atresia and restrictive VSD presenting with cyanosis from birth and underwent pulmonary valve perforation successfully.

**Conclusion:**

Duct-dependent pulmonary circulation is a pediatric emergency, palliative procedure for establishing adequate pulmonary blood flow is essential early in the life. In the management of duct-dependent pulmonary circulation, RVOT perforation is an effective and safe option in suitable high-risk subgroups. The induced pulmonary regurgitation along with established physiological antegrade flow would be beneficial in the remodeling of tripartite/hypertrophied small RV.

## Background

Hypoplastic right heart syndrome with pulmonary atresia is a rare form of cyanotic congenital heart disease with poor prognosis, high morbidity, and mortality. Early palliation for establishing adequate pulmonary flow is necessary for the relief of cyanosis and duct dependence. These patients are usually treated by aorto pulmonary shunt (BT shunt) followed by univentricular or 1.5 ventricular repair or biventricular repair at a later stage depending on the right ventricular growth if at all it grows. With the advent of use of radio frequency or laser-assisted perforation of pulmonary valve and balloon dilatation percutaneous techniques has garnered importance in achieving pulmonary flow. A series percutaneous techniques offered better outcome compared to surgical repair [1].

## Case presentation

**Fig. 1:**
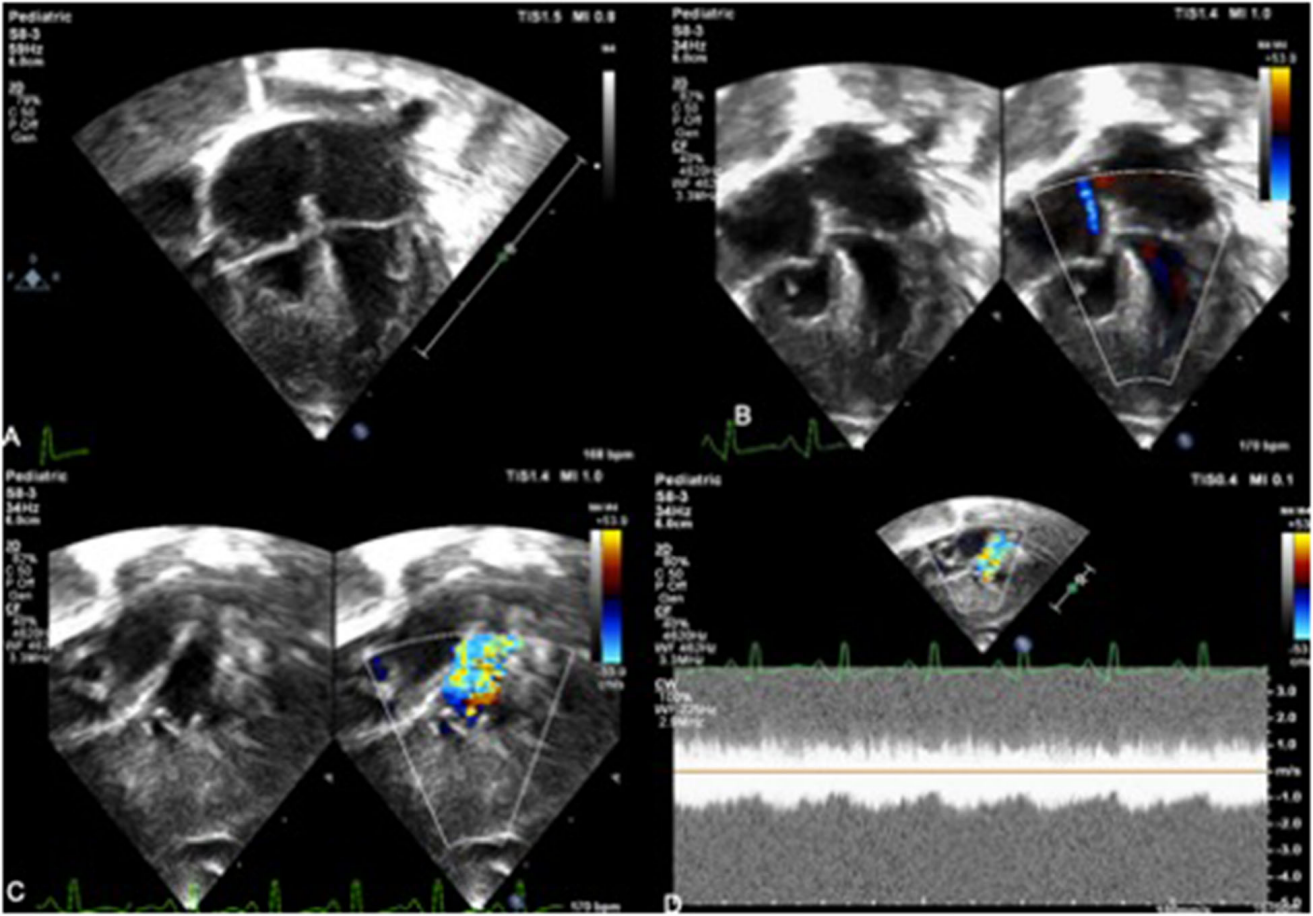
Echocardiogram showing hypoplastic and hypertrophied right ventricle with TV *Z* score-1.95. **b** Color doppler showing tricuspid regurgitation jet (RVSP 110 + mean RAP). **b**, **c** PDA seen filling the MPA. **d** CW showing the continuous flow across MPA due to its filling from PDA

A Neonate presented to us on day 1 of life with cyanosis, mild respiratory distress, and with a saturation of 70% in all 4 limbs. He was not evaluated for cardiac abnormalities during fetal life. He was born at 32 weeks of gestation and was weighing 2.1 kg. All the peripheral pulses are equally felt. There was a grade 3/6 pansystolic murmur at base of the heart. His electrocardiogram showed RV predominance with normal sinus rhythm and chest skiagram showed pulmonary oligemia with increased cardiothoracic ratio. Echocardiogram showed situs solitus, levocardia, atrioventricular, and ventriculoarterial concordance. Tricuspid valve leaflets had restricted movements with *z* score of annulus − 1.94. There was a severe TR, with measured RVSP of 110 mmHg (+mean RAP). There was a restrictive muscular ventricular septal defect, hypertrophied, hypoplastic tripartite RV, and valvular pulmonary atresia. The branch pulmonary arteries were confluent measuring 2.7/2.5 mm each. The duct arising from the under surface of the left aortic arch, supplying pulmonary circulation (as shown in Fig. 1).

**Fig. 2:**
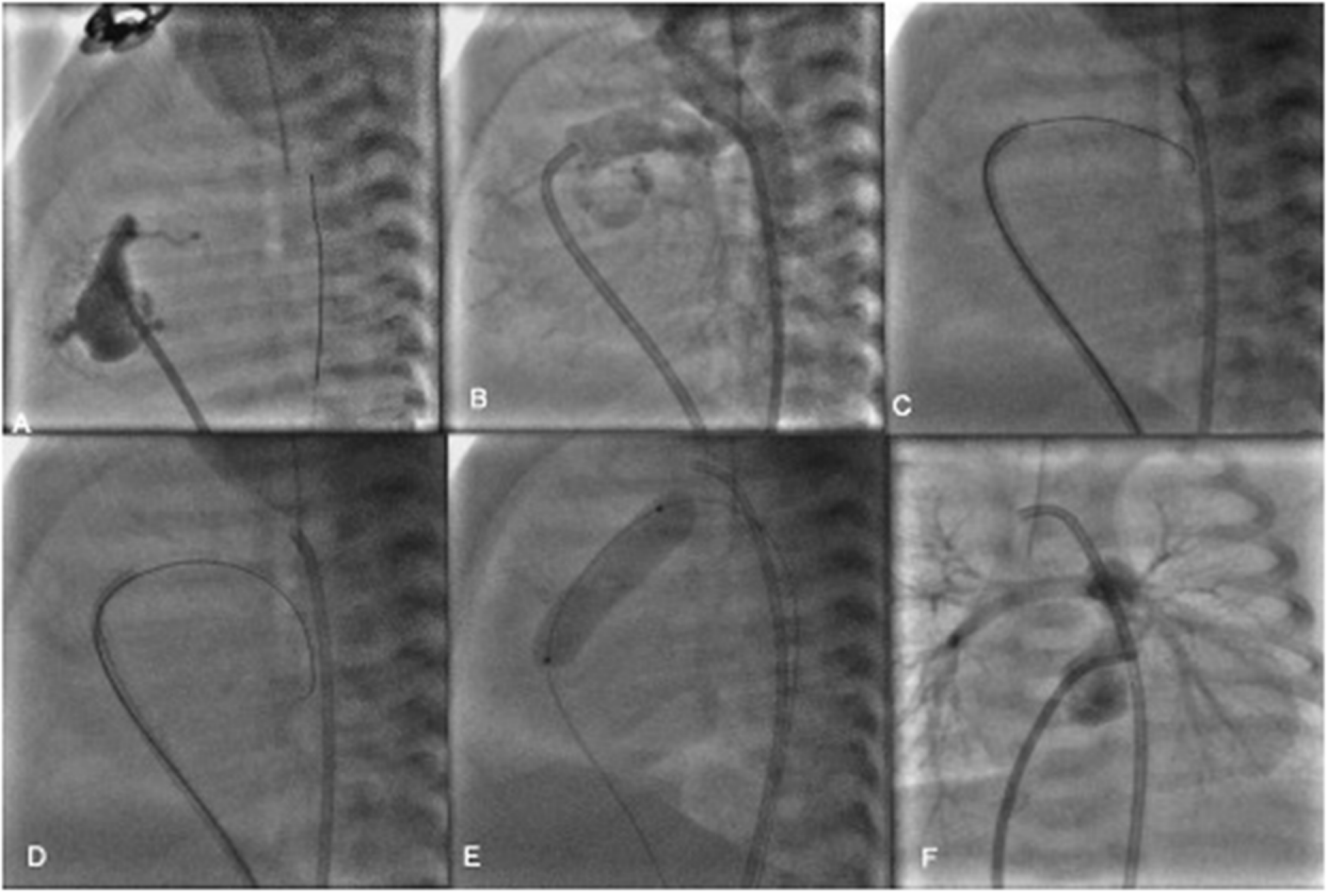
**a** JR guide at the atretic RVOT, with contrast filling of the RV and ending as bling pouch. **b** Injection in the arch of the aorta showing filling of the PA branches through PDA and also confirming guide position at the RVOT. **c** RVOT perforation with coronary guidewire. **d** Successful crossing of the guidewire across the atretic RVOT after perforation and wire parked in the DTA E Serial Balloon dilatation of the RVOT. **f** Final angiogram showing good antegrade pulmonary blood flow and arborization

**Fig. 3:**
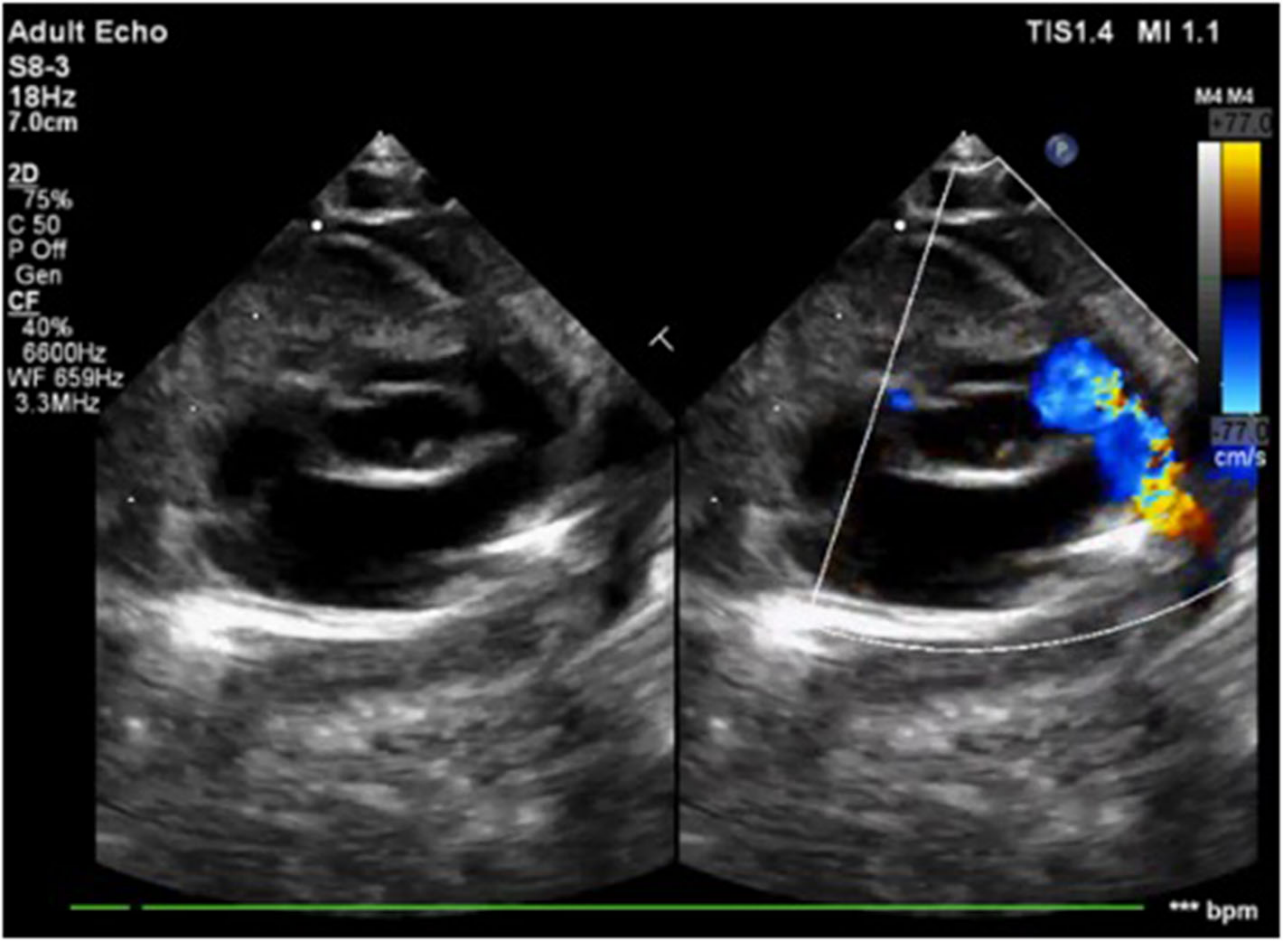
Post-procedure echocardiogram showing good antegrade flow across the pulmonary valve

He was immediately started on prostaglandin E1 infusion to maintain the patency of ductus arteriosus. After discussion in the combined meeting, neonate was considered to be ‘high-risk’ for BT shunt and option of PDA stenting was thought to be a safer option as stage I to ensure adequate pulmonary blood flow. Although, future single ventricular palliation was offered by the majority, some of us thought pulmonary valve perforation with induced pulmonary regurgitation would assist growth of this tripartite RV for future biventricular repair. Right femoral venous (6 Fr) and arterial lines (5 Fr) were established by digital palpation. A JR guiding catheter was crossed across the tricuspid valve and placed at the dimple of atretic pulmonary valve. The position was confirmed in orthogonal views with a hand injection in a diagnostic JR catheter placed in aortic arch. The course of the PDA showed the retrograde filling of MPA up to the atretic part reassuring the position of the guide. A GAIA (ASAHI^TM^ INTECC) wire was used to perforate the atretic pulmonary valve with serial dilatation using coronary balloons (4 × 12, 4.5 × 14, and 5 × 12) to obtain the desired diameter (as assessed preoperatively by echocardiogram). The final injection showed unobstructed contrast filling both branch pulmonary arteries with good bilateral arborization (as shown in Fig. 2a–f). The saturation increased from 70% to 94% with echo showing moderate pulmonary regurgitation, PDA still flowing (Fig. 3). The baby was managed with diuretics, extubated by 8 h. At 4 weeks follow-up, baby was doing fine with saturation of 87% although the right ventricle had not grown much. The duct had closed and RVSP by TR jet was 70+mean RA pressure.

## Discussion

Hypoplastic right heart syndrome (HRHS) is a rare cyanotic heart disease due to the underdevelopment of right heart structures (Tricuspid valve, RV, Pulmonary valve and pulmonary artery). The spectrum of disorders varies from simple hypoplastic RV with or without pulmonary stenosis to grossly underdeveloped RV with pulmonary and/or tricuspid atresia [1, 2]. Ventricular size can vary from grossly hypoplastic to normal-sized chambers [3]. HRHS is usually associated with ASD (atrial septal defect), VSD (ventricular septal defect), AVCD (atrioventricular cushion defect) and other complex defects. Clinical presentation depends on the extent of development of RV, pulmonary circulation, and usually present early in the life. The goals of early palliation include relief of cyanosis and duct dependence by establishing adequate pulmonary flow for development of right-sided structures. Treatment options include surgical systemic pulmonary shunt and percutaneous techniques like PDA stenting or pulmonary valve perforation. The difficulty in ductal stenting stems from the fact that ducts in these patients are tortuous and so incomplete stenting, stent migration can occur. Surgical correction at neonatal stage has higher mortality [4]. In the subset of patients with short-segment atresia management, options are well studied. Transcatheter pulmonary valve perforation offers a physiological approach for pulmonary flow and development of pulmonary arteries for future palliative surgeries. It also helps in the growth of the RV by the induced pulmonic regurgitation to a near normal size [3]. Also, the authors have experience of PV perforation and stenting of the RVOT in a sick infant (unpublished work); we felt that PV perforation offers a better palliative procedure short of surgery. The advantage with pulmonary valve perforation is that the pulmonary flow is more physiological and the induced pulmonary regurgitation helps in the growth of the right ventricle for future biventricular repair. In a series of 13 patients who underwent RVOT perforation, there was significant growth of the TV annulus and reduction in TR during at a mean follow-up of 13.2 months [5]. On the contrary there have been studies where there was no significant growth of the right ventricle following perforation [6–8]. The surgical aorto pulmonary shunting is a well-established palliative surgery in HRHS, but carries an increased risk in under-weight neonates. Complication with surgical shunting include pulmonary over-circulation, stenosis at the anastomosis site, asymmetric development of pulmonary vasculature, distortion of the vessels, nerve injury, and chylothorax [9]. In these patient’s assessment of the atretic pulmonary valve for its thickness, diameter of the RVOT, MPA, and branch PA’s is essential for selecting suitable patients. Perforation using hydrophilic coronary wires or radiofrequency ablation has been described in the literature [10–12]. Surgical correction (pulmonary valvotomy) at neonatal prove to be detrimental in some series [13]. Humpl et al. in their series of 50 patients of PA with IVS, attempted PV perforation in 30 patients by either stiff end of the coronary guide wire or RF ablation with a procedural success in 27 patients. They could find that with PV perforation though RV did not increase according to body growth at early follow up, it is adequate to maintain a biventricular circulation [10]. In a series of 33 patients of PA with IVS by Alwi et al. where RF ablation with balloon dilatation was compared with surgical valvotomy and BT shunt, percutaneous procedure was more efficacious and safe [1]. Procedural success has been described as establishment of adequate pulmonary flow, weaning from prostaglandin, and without requiring reintervention [8]. Complications are rare including perforation with tamponade, arrhythmia, and vascular injury [10]. Surgical correction (pulmonary valvotomy) at early stage prove to be detrimental in some series [13]. In our case, we proceeded with percutaneous intervention, as less invasive procedure in a sick neonate is probably safer. However, the child succumbed to pneumonia-sepsis after 5 weeks of procedure.

## Conclusion

Duct-dependent pulmonary circulation is a pediatric emergency, palliative procedure for establishing adequate pulmonary blood flow is essential early in the life. In the management of duct-dependent pulmonary circulation, RVOT perforation is an effective and safe option in suitable high-risk subgroups. The induced pulmonary regurgitation along with established physiological antegrade flow would be beneficial in the remodelling of tripartite/hypertrophied small RV.

## Supplementary information

**Additional file 1:.**

**Additional file 2:.**

**Additional file 3:.**

**Additional file 4:.**

**Additional file 5:.**

**Additional file 6:.**

**Additional file 7:.**

## Data Availability

The datasets used and/or analyzed during the current study are available from the corresponding author on reasonable request.

## Abbreviations

HRHS: Hypoplastic right heart syndrome
PV: Pulmonary valve
VSD: Ventricular septal defect
ASD: Atrial septal defect
AVCD: Atrioventricular cushion defect
PDA: Patent ductus arteriosus
RV: Right ventricle
RVOT: Right ventricular outflow tract
MPA: Main pulmonary artery
JR: Judkin’s right
Fr: French units
